# Patients with chronic autoimmune demyelinating polyneuropathies exhibit cognitive deficits which might be associated with CSF evidence of blood-brain barrier disturbance

**DOI:** 10.1371/journal.pone.0228679

**Published:** 2020-02-04

**Authors:** Yavor Yalachkov, Valerie Uhlmann, Johannes Bergmann, Dilara Soydaş, Stefan Frisch, Marion Behrens, Christian Foerch, Johannes Gehrig

**Affiliations:** 1 University Hospital Frankfurt, Department of Neurology, Frankfurt am Main, Germany; 2 Institute of Psychology, Goethe-University, Frankfurt am Main, Germany; Hannover Medical School, GERMANY

## Abstract

**Background:**

Chronic autoimmune demyelinating polyneuropathies (CADP) result in impaired sensorimotor function. However, anecdotal clinical observations suggest the development of cognitive deficits during the course of disease.

**Methods:**

We tested 16 patients with CADP (11 patients with chronic inflammatory demyelinating polyneuropathy, 4 patients with multifocal motor neuropathy and 1 patient with multifocal acquired demyelinating sensory and motor neuropathy) and 40 healthy controls (HC) with a neuropsychological test battery. Blood-brain-barrier dysfunction (BBBd) in patients was assessed retrospectively by analysing the cerebral spinal fluid (CSF) status at the time the diagnosis of CAPD was established.

**Results:**

CADP patients failed on average in 1.7 out of 9 neuropsychological tests (SD ± 1.25, min. 0, max. 5). 50% of the CADP patients failed in at least two neuropsychological tests and 44.3% of the patients failed in at least two different cognitive domains. CADP patients exhibiting BBBd at the time of first diagnosis failed in more neuropsychological tests than patients with intact integrity of the BBB (p < 0.05). When compared directly with the HC group, CADP patients performed worse than HC in tests measuring information processing ability and speed as well as phonemic verbal fluency after adjusting for confounding covariates.

**Conclusions:**

Our results suggest that mild to moderate cognitive deficits might be present in patients with CAPD. One possible tentative explanation, albeit strong evidence is still lacking for this pathophysiological mechanism, refers to the effect of autoimmune antibodies entering the CNS via the dysfunctional blood-brain barrier typically seen in some of the CADP patients.

## Introduction

Chronic autoimmune-mediated demyelinating polyneuropathies (CADP) such as chronic inflammatory demyelinating polyneuropathy (CIDP), multifocal acquired demyelinating sensory and motor neuropathy (MADSAM) or multifocal motor neuropathy (MMN) affect the peripheral nervous system (PNS), presumably via an antibody-mediated destruction of the myelin sheath of the peripheral nerves [1], causing sensorimotor symptoms. Some clinical observations suggest, however, that cognitive deficits might develop during the course of disease, too. In a preliminary analysis with a sample size of 7 CIDP patients executive function, selectiveness and divisibleness of attention were significantly lower as compared to healthy controls [2]. In another study, 34.1% of the included 41 CIDP patients reported subjective memory deficits but the average Mini-Mental State Examination score (MMSE) was within normal range [3]. A case series reported that a small number of patients vaccinated with the OspA antigen of Borrelia burgdorferi have developed MMN, CIDP, cognitive deficits or even a combination of both CIDP and cognitive deficits, suggesting that some common autoimmune-mediated mechanisms might underlie both peripheral and central nervous system (CNS) damage [4]. Another case report described a manifestation of CIDP and an additional cognitive impairment in a 60-year-old patient with a rapid cognitive improvement after intravenous immunoglobulin treatment [5]. Blood-brain barrier dysfunction (BBBd) can be seen in CADP patients [6] and might theoretically constitute a means for antibodies to enter the CNS, although there is no strong evidence for this mechanism yet.

There is no rigorous scientific data supporting the notion of cognitive deficits in CADP and no rational pathophysiological mechanism has been identified so far. In our study, we compared the neuropsychological performance of CADP patients to established test-specific norms in several cognitive domains. Additionally, we compared the patients’ performance in each neuropsychological test with a group of healthy controls (HC) after adjusting for confounding variables. Last but not least, since experimental and observational studies have suggested a link between autoimmune-mediated BBBd and cognitive deficits [7–9], we investigated the association between the integrity of the BBB (as measured by the cerebrospinal fluid (CSF)/serum albumin quotient determined during the time of first CAPD diagnosis) and current cognitive performance.

## Materials and methods

### Study population

16 patients with CADP (11 patients with CIDP, 1 patient with MADSAM, 4 patients with MMN) were included in the study. Patients were recruited via the neurology department at the University Hospital in Frankfurt am Main, Germany and gave an informed consent. The ethics committee of the University of Frankfurt Medical Faculty approved this study.

Further clinical characteristics of the patients can be found in Table 1. Diagnoses of “definite”, “probable” or “possible” CIDP/MMN were determined according to the European Federation of Neurological Societies/Peripheral Nerve Society (EFNS/PNS) criteria [10]. Further characteristics such as proximal/distal affection, CNS and other comorbidities, central demyelination in magnetic resonance imaging (MRI), antibody testing results, CSF/serum results, subjective reports on neuropathic pain, current and previous immunomodulatory treatments were extracted from the individual patient’s medical history.

**Table 1:** Clinical data of the CADP cohort.

	diagnosis according to EFN criteria	proximal affection	distal affection	CNS comorbidities	other comorbidities	central demyeli-nation in MRI ^a^	antibodies ^b^	Q (CSF/Ser) x [10x-3] ^c^	age-dependent cut-off ^d^	BBB dysfunction ^e^	intrathecal Ig synthesis in Reiber’s diagram ^f^	oligoclonal banding ^g^	report on current neuropathic pain ^h^	current treatment ^i^	previous treatments ^j^
**P01**	definite CIDP	yes	yes	no	-	no	GM-1-ab (-)	8.50	5.93	yes	negative	negative	pain in the hands	IVIG	-
**P02**	definite CIDP	yes	yes	no	high blood pressure	no MRI available	data not available	data not available		yes	data not available	negative	no	IVIG	azathioprine corticosteroids
**P03**	definite CIDP	yes	yes	no	high blood pressure, coronary heart disease, peripheral arterial occlusive disease, impaired glucose tolerance, smoking	no MRI available	GM-1-ab (-)	8.80	8.53	yes	negative	not performed	right-side gluteal pain	IVIG	corticosteroids
**P04**	definite CIDP	no	yes	no	high blood pressure, cardiac pacemaker, pulmonary embolism, ex-smoker	no MRI available	data not available	9.70	8.47	yes	negative	negative	no	IVIG	corticosteroids
**P05**	definite CIDP	no	yes	no	smoking	no	GM1-ab (-) MAG-ab (-)	6.30	7.67	no	negative	negative	no	IVIG	corticosteroids
**P06**	definite CIDP	yes	yes	no	inguinal hernia, lumbar disc herniation	no	GM1-ab (-)	11.10	8.80	yes	negative	not performed	no	IVIG	corticosteroids
**P07**	definite CIDP	yes	yes	no	Waldenström's macroglobulinemia	no MRI available	MAG-ab (-)	14.40	8.60	yes	negative	not performed	no	IVIG	bendamustine rituximab IVIG azathioprine
**P08**	definite MMN	yes	yes	no	ex-smoker	no	GD1a-ab (+) MAG-ab (-)	4.90	6.60	no	negative	not performed	no	IVIG	-
**P09**	definite MMN	no	yes	no	polytrauma in juvenile years	no MRI available	GM1-ab (+)	5.60	8.93	no	negative	not performed	no	rituximab	IVIG
**P10**	definite CIDP	yes	yes	no	coronary heart disease, coronary bypass surgery, prostatic hyperplasia, hearing impairment left ear	no MRI available	MAG-ab (-)	data not available		data not available	data not available	data not available	no	IVIG	
**P11**	probable CIDP	no	yes	no	thrombocytopenia, dermatitis, COPD, prostate cancer	no	GM2-ab (+)	11.10	8.60	yes	negative	not performed	no	cortico- steroids	corticosteroids
**P12**	definite MMN	no	yes	no	high blood pressure, idiopatic hyper-CK-emia, ex-smoker, former alcohol abuse	no	GM1-ab (+)	4.90	7.27	no	negative	not performed	no	IVIG	-
**P13**	definite CIDP	yes	yes	no	high blood pressure, lumbar disc herniation	no MRI available	data not available	4.00	6.87	no	negative	not performed	no	IVIG	corticosteroids azathioprine methotrexate
**P14**	definite MMN	no	yes	no	ex-smoker	not in the cervical MRI	GM1-ab (+)	4.20	7.27	no	negative	not performed	no	IVIG	-
**P15**	definite CIDP (atypical CIDP, MADSAM)	yes	yes	no	high blood pressure, monoclonal gammopathy of undetermined significance	no	GM1-ab (+) GD1a-ab (+) GD1b-ab (+) asialoganglioside-ab (+) MAG-ab (-)	5.70	6.67	no	negative	not performed	no	IVIG	-
**P16**	definite CIDP	yes	yes	no	-	no MRI available	data not available	9.00	7.27	yes	negative	not performed	pain in the calves	IVIG	-

^**a**^ “no” = no central demyelination in MRI/according to the MRI reports/medical history. “not in the cervical MRI” = one patient had no cranial MRI but only cervical MRI available; “no MRI available” = no MRI reports/imaging were available for the respective patient.

^**b**^ (+) = positive results for the respective antibody (ab); (-) = negative results for the respective antibody according to patients’ medical history. “Data not available” = no reports on antibody testing were found in the respective medical history.

^**c**^ Cerebrospinal fluid/serum albumin quotient. “Data not available” = no reports were found in the patient’s medical history.

^**d**^ Individual age-dependent cut-off albumin quotient above which a BBBd can be assumed.

^**e**^ blood-brain barrier dysfunction (“yes” = present; “no” = not present). For P02 only the descriptive results from the medical reports were available, confirming a BBBd without describing the exact CSF/serum albumin quotients.

^**f**^ intrathecal Ig synthesis according to the Reiber’s diagram.

^**g**^ “negative” = no difference between CSF and serum with regard to oligoclonal bands detected; “not performed” = no testing for oligoclonal bands was performed during the diagnostic work-up.

^**h**^ subjective reports on current neuropathic pain (“no” = no pain reported).

^**i + j**^ current and previous immunomodulatory treatment. “-”= none. “IVIG” = intravenous immunoglobulin therapy.

To further corroborate our findings from comparing the patients’ performance with the respective reference means in the neuropsychological tests, we conducted an additional analysis, where we compared patients’ performance directly with the performance of healthy controls. The data of the HC control group has been analysed in a previous study of our group [11] with a different focus and patient population, and should serve here only as part of the further analysis of the CADP population.

The average age of the CADP group (3 female, 13 male) was 61.5 years (standard deviation [SD] 14.81 years). Their average INCAT ODSS score was 3.63 (SD 2.13). There was no previous history of reported or clinically detected cognitive deficits in any of the patients. The average age of the HC group (30 female, 10 male) was 41.5 years (SD 14.69). Table 2 illustrates the remaining descriptive statistics for CADP and HC.

**Table 2:** Descriptive statistics of the chronic autoimmune demyelinating polyneuropathy (CADP) patients and healthy controls (HC). $\bar{x}$ = mean; SD = standard deviation; BDI-score = Beck Depression Inventar score; VAS relative score = relative score in Visual Analogue Scale; RCFT IR = Immediate Recall trial in the Rey Complex Figure Test; SDMT = Symbol Digit Modalities Test; VLMT total = total number of correctly recalled items in trials 1 to 5 of the Verbaler Lern- und Merkfähigkeitstest; VLMT 5–7 = “trial 7”–“trial 5” difference in the VLMT; PASAT = Paced Auditory Serial Addition Test; TMT = Trail Making Test; RWT p/s = phonemic/semantic subtests of the Regensburger Wortflüssigkeits-Test; 9-HPT = 9-hole peg test; WST-z-score = Wortschatztest z-score.

		n	$\bar{x}$	SD	95% confidence interval	
					lower bound	upper bound
age	HC	n = 40	41.45	14.69	36.75	46.15
	CADP	n = 16	61.50	14.81	53.61	69.39
highest degree of education: school	HC	n = 2				
	CADP	n = 0				
highest degree of education: school and professional training	HC	n = 3				
	CADP	n = 11				
highest degree of education: university	HC	n = 35				
	CADP	n = 5				
BDI score	HC	n = 40	3.58	4.40	2.13	5.03
	CADP	n = 16	9.06	6.60	5.55	12.58
VAS relative score	HC	n = 40	-0.03	0.21	-0.10	0.03
	CADP	n = 16	-0.09	0.25	-0.22	0.04
RCFT IR	HC	n = 38	22.60	5.44	20.86	24.34
	CADP	n = 16	18.31	6.61	14.79	21.83
SDMT	HC	n = 40	54.65	10.61	51.26	58.04
	CADP	n = 16	41.38	9.40	36.37	46.39
VLMT total	HC	n = 40	60.33	7.97	57.78	62.87
	CADP	n = 16	50.81	11.39	44.74	56.88
VLMT 5–7	HC	n = 40	0.60	1.57	0.1	1.10
	CADP	n = 16	2.06	1.73	1.14	2.99
PASAT	HC	n = 39	9.41	6.73	7.23	11.59
	CADP	n = 16	18.50	14.95	10.53	26.47
TMT-A	HC	n = 40	26.73	8.61	23.97	29.48
	CADP	n = 16	42.31	13.08	35.34	49.28
TMT B/A	HC	n = 40	2.27	0.67	2.06	2.49
	CADP	n = 16	2.09	0.67	1.73	2.45
RWTp	HC	n = 40	26.25	6.25	24.25	28.25
	CADP	n = 16	20.56	6.53	17.08	24.04
RWTs	HC	n = 40	39.60	9.40	36.60	42.61
	CADP	n = 16	35.88	8.12	31.55	40.20
9-HPT dominant hand	HC	n = 40	17.80	3.33	16.73	18.86
	CADP	n = 16	29.43	11.55	23.28	35.59
WST z-score	HC	n = 40	0.78	0.55	0.60	0.95
	CADP	n = 16	0.40	0.69	0.03	0.77

### Data collection

Participants completed a neuropsychological test battery consisting of pre-task Visual Analogue Scale (VAS 1) on subjective performance capability, Rey Complex Figure Test (RCFT) [12], Symbol Digit Modalities Test (SDMT) [13], Verbaler Lern- und Merkfähigkeitstest (VLMT, a German adaptation of the Rey Auditory Verbal Learning Test) [14], Paced Auditory Serial Addition Test (PASAT) [15], Trail Making Test (TMT) [16], Regensburger Wortflüssigkeits-Test (RWT) [17], Wortschatztest (WST, a German vocabulary test) [18], Beck Depression Inventory (BDI) [19], 9-hole-Peg-test (9-HPT) and post-task Visual Analogue Scale (VAS 2) on subjective performance capability to evaluate changes in fatigue related to the neuropsychological testing.

The presence of blood-brain-barrier dysfunction (BBBd) at the time of first CAPD diagnosis was determined retrospectively by comparing each patient’s albumin quotient (CSF/serum albumin index) derived from the CSF/serum analysis at the time of first diagnosis with the respective age-dependent cut-off albumin quotient. The CSF and serum albumin determinations had been carried out during a routine diagnostic work up of autoimmune-mediated polyneuropathies, i.e. there was no delay more than a couple of minutes between the two examinations. Those data were accessed retrospectively via the patients’ medical records.

### Statistical analysis

We concentrated on cognitive domains usually affected in patients with chronic neurological autoimmune diseases [20]: general information processing ability (measured by SDMT); information processing speed and flexibility (measured by PASAT, TMT-A and TMT B/A); learning and memory, i.e. learning capacity (measured by the total number of correctly recalled items in trials 1 to 5 of the VLMT [VLMT total]) and memory loss due to forgetting over time (indicated by the “trial 7”–“trial 5” difference in the VLMT [VLMT 5–7]); visuospatial recall memory (measured by the Immediate Recall RCFT trial, RCFT_IR); phonemic and semantic verbal fluency (phonemic and semantic subtests of the RWT [RWTp and RWTs]). Cognitive impairment was indicated by the number of neuropsychological tests, in which patients’ performance was more than one standard deviation (SD) below the respective reference mean. This approach is well-established and often used in the field of cognitive research in multiple sclerosis and clinical neuropsychology [21–25]. The threshold for defining cognitive impairment varies from study to study, ranging usually from one to two SD below the reference mean. Because of the rather exploratory nature of our study and the limited sample size, we decided to use one SD below the mean as a threshold, in order to optimize the power of the study. Grouping all subjects with performance worse than one SD together might result in grouping together mildly and severely impaired patients but this is a reasonable risk for an exploratory study and has been successfully used before for cognitive research in neurological patients [21,22,24–29]. For clarity and comprehensibility reasons, we will refer to performance more than one SD below the respective reference mean in a neuropsychological test as a “failed test”, while we point out in the discussion that defining the threshold at lower z-scores would lead automatically to a different prevalence of cognitive impairment. Additionally, the percentage of patients who exhibited impairment in at least two from the five studied domains (general information processing ability; information processing speed and flexibility; learning and memory; visuospatial recall memory; verbal fluency) was computed. This decision was motivated again by the field of cognitive research in multiple sclerosis where usually not only the absolute number of tests with performance below the respective reference mean is computed but also the proportion of the affected cognitive domains is taken into consideration [22,26]. Task-related changes in fatigue were measured with the relative VAS score. The relative VAS score indicates how much the fatigue has increased during the neuropsychological testing relatively to the individual baseline score and was computed following the formula (VAS 2—VAS 1)/VAS 1. Premorbid intelligence and cognitive reserve were evaluated by means of calculating patients’ years of education and vocabulary (Z-scores for WST) as suggested by Sumowski et al. [30]. Depression was measured with BDI.

The role of BBBd at the time of first diagnosis was tested with a multivariate ANOVA (MANOVA) on the data of the CADP patients, where patients with BBBd were compared to patients with an intact BBB (BBBi) with respect to their number of failed neuropsychological tests, age, years of education, vocabulary, depression, task-related changes in fatigue, manual dexterity, time between the CSF analysis and neuropsychological testing as well as the INCAT ODSS score. The INCAT ODSS (Inflammatory Neuropathy Cause and Treatment Overall Disability Sum Score) was measured at the time of cognitive testing. Additionally, correlations between the BBB integrity (dysfunctional/intact) and the presence of proximal/distant sensorimotor affection [Cramér's V/Phi coefficients] as well as between the extent of BBBd (as measured by the CSF/serum albumin quotient) and the degree of cognitive impairment (as measured by the number of failed tests) [Pearson correlation] were calculated.

Since BBBd has been shown mostly for CIDP but our sample included also patients with MMN and MADSAM, we decided to examine whether our observations would still hold true if only patients with CIDP are included in the BBB analysis. A separate likelihood-ratio analyses as well as Cramér's V/Phi coefficients were computed to test for association between a BBBd and cognitive impairment indicated by failure in at least two neuropsychological tests.

Neuropsychological performance of CADP patients and HC as well as their task-related changes in fatigue were compared with a MANOVA, where age, years of education, WST-Z-score and BDI-score served as covariates, thus ensuring that any group differences are adjusted for the confounding influence of age, education/premorbid intelligence and depression on the neuropsychological performance. Additionally, patients’ performance in TMT-A, where restricted motor functions of the dominant hand due to sensorimotor symptoms of the neuropathy might be a confounding variable, was compared with the performance of HC by calculating an additional ANOVA with the above-mentioned covariates and including the average 9-HPT time for the dominant hand as an additional covariate.

## Results

CADP patients’ mean number of failed neuropsychological tests (i.e. performance > 1 SD below the reference mean) was 1.7 (SD ± 1.25, min. 0, max. 5). In each of the neuropsychological tests there was at least 1 from 16 (6%) CADP patients who showed a test-specific impairment, i.e. performance of at least 1 SD below the reference mean. In some of the tests this percentage reached remarkable levels (e.g. 5 from 16 CADP patients [31%] in PASAT and TMT-A or 4 from 16 patients [25%] in VLMT 5–7). 50% of the patients (8 from 16) failed in at least two neuropsychological tests. 44% of the patients (7 from 16) exhibited cognitive impairment in at least two different cognitive domains (Table 3).

**Table 3:** Neuropsychological performance of chronic autoimmune demyelinating polyneuropathy (CADP) patients. SD = standard deviation; RCFT IR = Immediate Recall trial in the Rey Complex Figure Test; SDMT = Symbol Digit Modalities Test; VLMT total = total number of correctly recalled items in trials 1 to 5 of the Verbaler Lern- und Merkfähigkeitstest; VLMT 5–7 = “trial 7”–“trial 5” difference in the VLMT; PASAT = Paced Auditory Serial Addition Test; TMT = Trail Making Test; RWT p/s = phonemic/semantic subtests of the Regensburger Wortflüssigkeits-Test. CI = cognitive impairment.

Cognitive domain	Neuropsychological test	Number of patients with performance > 1 SD below the average	% of patients with performance > 1 SD below the average
General information processing ability	SDMT	3	19
Information processing speed and flexibility	PASAT	5	31
	TMT-A	5	31
	TMT B/A	1	6
Learning and memory	VLMT total	3	19
	VLMT 5–7	4	25
Visuospatial recall memory	RCFT_IR	2	13
Verbal fluency	RWTp	2	13
	RWTs	2	13
Cognitive impairment (CI)	CI in ≥ 2 tests	8	50
	CI in ≥ 2 domains	7	44

**Fig 1:**
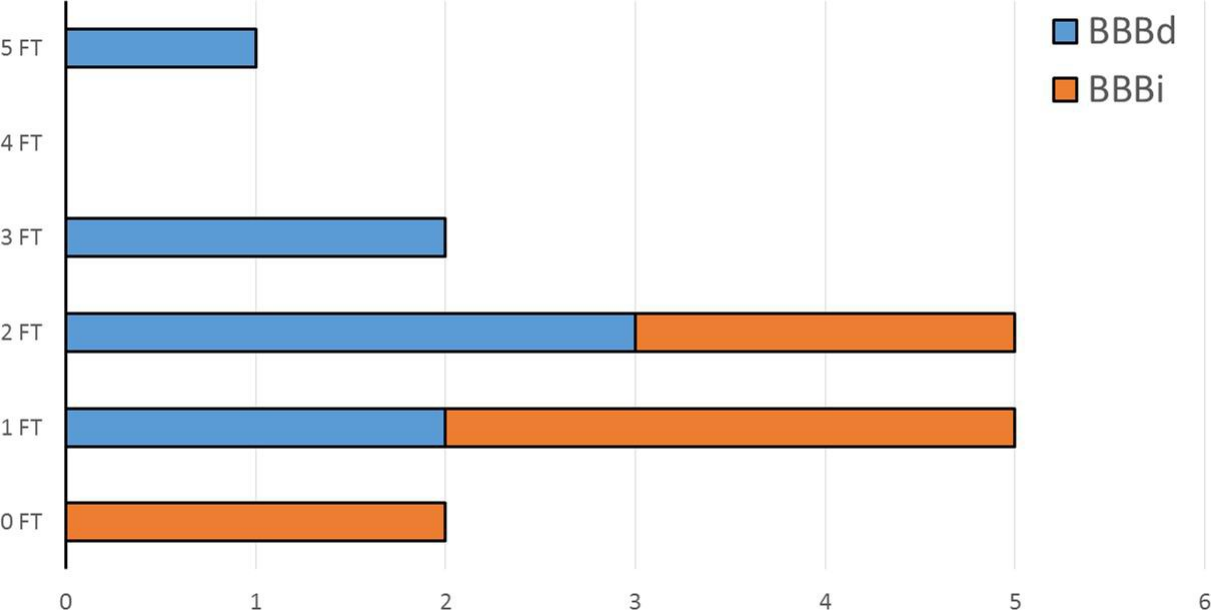
The y-axis shows the distribution in the number of the failed (i.e., performance > 1 SD below the respective reference mean) neuropsychological tests (min. 0, max. 5, FT = failed test, i.e. below average performance). The x-axis illustrates the number of patients according to this distribution. The CADP patients with an intact BBB (BBBi) are shown in orange and the patients with a BBB dysfunction (BBBd) are shown in blue.

The results of the CSF analysis of one of the patients were not available. Eight from the remaining 15 CADP patients (53%) exhibited BBBd at the time of first CADP diagnosis (mean range between CSF analysis and current neuropsychological testing 4.1 years, SD 3.1 years) but none exhibited CSF pleocytosis or immunoglobulin synthesis. Artificial blood contamination of the CSF was present in only two patients: one patient had only 1 erythrocyte/μl and a dysfunctional BBB, while another had 132 erythrocytes/μl and an intact BBB. When comparing both groups (BBBi vs. BBBd) with the MANOVA, the only significant group difference was with regard to the number of failed neuropsychological tests (BBBi’s mean number of failed tests 1.0, SD 0.8 vs. BBBd’s mean number of failed tests 2.4, SD 1.3, p = 0.038, Table 4, Fig 1). There was no significant group difference with regard to age, years of education (non-parametric Mann-Whitney-test, mean ranks), BDI, VAS relative score, 9-HPT, WST-Z-score, time between the CSF analysis and cognitive testing as well as INCAT ODSS score (Table 4). The general influence of the group factor on all nine dependent variables was not significant (F-value 0.67, p > 0.05). There was no significant correlation between proximal affection and BBB integrity (Cramér's V/Phi coefficient φc/ rφ = 0.327, p = 0.205). No correlation could be computed between the presence of distal affection and BBB integrity since all the subjects exhibited a distal affection. Individual z-score profiles of the CADP patients are provided in Table 5.

**Table 4:** Neuropsychological performance of CADP patients with intact and dysfunctional blood-brain barrier. BDI = Beck Depression Inventory; 9-HPT = 9-hole-Peg-test; WST-z = Wortflüssigkeitstest Z-score; Δ LP-CT = time between the lumbar puncture and cognitive testing in years; INCAT ODSS = INCAT Overall Disability Sum Score; BBB = blood-brain-barrier; IV = independent variable; DV = dependent variable; n.s. = not significant; ⁰ = mean rank; ⁰⁰ = non-parametric Mann-Whitney-test. n.s = not significant (p > 0.05).

DV	Blood-brain barrier (BBB) at time of first diagnosis					
	BBB intact (n = 7; 2 females)		BBB dysfunctonal (n = 8; 1 female)		F-value for the IV group on this DV in the MANOVA	Group difference in the MANOVA (between-subjects effect)
	mean	SD	mean	SD		
age	**53,7**	*12*,*4*	**65,6**	*13*,*8*	2,3	n.s.
years of education	*non-parametric Mann-Whitney-test*				0,3	n.s.
BDI	**12,3**	*8*,*6*	**6,6**	*3*,*4*	3,8	n.s.
VAS relative score	**0,0**	*0*,*1*	**-0,1**	*0*,*3*	0,6	n.s.
9-HPT	**33,8**	*15*,*0*	**26,7**	*7*,*3*	1,1	n.s.
WST-z	**0,2**	*0*,*7*	**0,5**	*0*,*7*	1,0	n.s.
Δ LP-CogTest	**3,9**	*3*,*9*	**4,3**	*2*,*4*	0,1	n.s.
INCAT ODSS	**4,0**	*2*,*7*	**3,3**	*1*,*8*	0,3	n.s.
number of failed tests	**1,0**	*0*,*8*	**2,4**	*1*,*3*	5,5	p = 0.038

**Table 5:** Individual z-score profiles of the chronic autoimmune demyelinating polyneuropathy (CADP) patients. RCFT IR = Immediate Recall trial in the Rey Complex Figure Test; SDMT = Symbol Digit Modalities Test; VLMT total = total number of correctly recalled items in trials 1 to 5 of the Verbaler Lern- und Merkfähigkeitstest; VLMT 5–7 = “trial 7”–“trial 5” difference in the VLMT; PASAT = Paced Auditory Serial Addition Test; TMT = Trail Making Test; RWT p/s = phonemic/semantic subtests of the Regensburger Wortflüssigkeits-Test. BBBd = blood-brain-barrier dysfunctional; BBBi = blood-brain-barrier intact.

		SDMT	PASAT	TMT A	TMT B/A	VLMT total	VLMT 5–7	RCFT IR	RWTp	RWTs
**BBBd**	**P01**	-0.38	0.81	-0.52	0.39	1.86	-0.20	0.7	-1.282	-1.282
	**P02**	-0.26	-1.67	-0.84	-0.84	-0.57	-1.34	-0.4	0.674	1.282
	**P03**	-0.76	0.88	-1.28	1.04	-1.18	-1.34	-3.8	-1.282	0.994
	**P04**	-1.55	-2.67	-0.25	0.00	1.24	0.70	1.3	0.994	-0.674
	**P06**	-0.15	-1.44	-0.84	-0.67	0.53	-0.32	2.5	0.674	1.282
	**P07**	0.74	-0.20	-1.28	1.28	-1.48	-1.34	1.9	0	1.282
	**P11**	-1.02	-1.36	-0.25	-0.39	-0.37	-2.35	0.8	0.674	0.674
	**P16**	0.5	-0.35	-1.28	1.28	0.74	-0.19	-0.9	-0.994	1.282
**BBBi**	**P05**	1.14	-0.59	-1.28	0.52	0.23	-0.32	-0.8	-0.674	0.994
	**P08**	-0.06	0.81	0.52	1.04	0.74	-0.20	-0.5	-0.674	0
	**P09**	-0.48	-0.89	0.00	-0.39	-1.18	0.70	-0.5	-0.994	-1.282
	**P12**	-0.15	1.19	0.00	-0.39	0.94	-0.19	1	0	0
	**P13**	-1.03	1.19	-0.25	-0.39	2.35	0.70	2.6	0	0.674
	**P14**	-0.62	0.03	0.52	-1.65	1.14	-0.32	1.3	-0.994	0.674
	**P15**	-0.38	0.44	-1.28	1.65	0.46	-0.40	-1.9	-0.674	-0.994

There was a significant correlation between the extent of BBBd (as measured by the CSF/serum albumin quotient) and the cognitive impairment (as measured by the number of failed tests) (r = 0.53, p = 0.026, n = 14, one-tailed Pearson correlation test).

When analysing only the CIDP patients (n = 10), we observed that four of them exhibited cognitive impairment in only one neuropsychological test. 50% of these four subjects (2 from 4) had an intact BBB, while 100% (6 from 6) of the subjects who failed in two or more neuropsychological tests showed a BBBd at the time of their first diagnosis (likelihood quotient for a likelihood ratio test 4.5, p = 0.035). Thus, there was still a strong association between the presence of a BBBd and a later cognitive impairment (Cramér's V/Phi coefficient φ_*c*_/ r_φ_ 0.61, p = 0.05).

When comparing CADP patients directly to HC in the complementary analysis, the MANOVA showed a significant group difference only for TMT-A and RWTp but not for the other neuropsychological tests or for task-related fatigue change (F-values 7.2 and 4.2, p < 0.05, adjusted for covariates age, years of education, WST-Z-score and BDI-score). The general influence of the group factor on all neuropsychological tests was not significant (F-value 1.02, p > 0.05). Additionally, patients’ performance in TMT-A was compared to the performance of HC with an ANOVA, where the average 9-HPT time for the dominant hand was included as a covariate in addition to the above-mentioned covariates to control for confounding manual dexterity restrictions. The group difference between CADP and HC with regard to TMT-A remained significant (F-value 5.0, p < 0.05, adjusted for covariates age, years of education, WST-Z-score and BDI-score, 9-HPT).

## Discussion

CADP patients exhibited mild to moderate cognitive deficits, failing on average in 1.7 from the 9 analysed neuropsychological tests. One-fourth to one-third of the sample (25% and 31%, respectively) performed worse than the reference means in tests measuring general information processing ability, speed and flexibility as well as learning and memory. Remarkably, half of the patients failed in at least two neuropsychological tests and almost half of the sample failed in at least two different cognitive domains. Cognitive deficits were associated with the presence of a BBBd at the time of first CADP diagnosis and this remained true even in a subsample of CIDP patients only.

In direct comparison of the neuropsychological performance of CADP patients with HC, we adjusted the results for confounding variables such as age, years of education, manual dexterity etc. by including these variables as covariates in the (M)ANOVA analyses. CADP performed more poorly in tests measuring information processing ability and speed as well as phonemic verbal fluency even after adjusting for the influence of the confounding variables.

The finding that cognition is impaired in CADP patients is surprising since CADP usually affect the PNS and not the CNS. However, the traditional view that inflammatory conditions of the central and peripheral nervous system are restricted to either compartment of the nervous system has been recently challenged, e.g. by studies reporting an involvement of peripheral nerves in MS patients [31–33]. One conceivable hypothesis is that antibodies cross the dysfunctional BBB and unfold demyelinating effects in the CNS. While there is no strong evidence for this mechanism, one possible hint at its significance might be the findings that patients with BBBd at the time of first CAPD diagnosis are more likely to develop cognitive impairment when tested with a broad neuropsychological battery and that the extent of BBBd correlates with the degree of cognitive deficits. Indeed, case reports and studies of Guillain Barré Syndrome (GBS), an acute demyelinating polyneuropathy, have revealed evidence for CNS involvement reflected by neuropsychiatric manifestations [34,35]. In one of these studies the CSF protein concentration correlated with the severity of psychosis observed in GBS [34]. Studies with other patient populations and disease models have also suggested a link between autoimmune-mediated BBBd and cognitive/neuropsychiatric deficits (and thus a CNS involvement) [7–9]. Findings from the field of rheumatoid arthritis (RA) show that CNS involvement in RA occurs possibly due to BBBd associated with chronic inflammation and that circulating immune complexes may cause neuroinflammatory responses in the brain [36]. Indeed, Sağ et al. demonstrated that serum levels of S100β and GFAP, two brain-specific proteins which are usually elevated in blood when the BBB is damaged, were significantly higher in RA patients compared to HC [36]. Perhaps through a similar mechanism, concomitant BBBd and circulating autoimmune agents might mediate the development of cognitive deficits in CADP. However, the nature of this pathophysiological mechanism remains inconclusive as long as there are no prospective, well-controlled longitudinal studies.

An intriguing question is why the cognitive deficits in CADP have not become clinically more apparent yet. The difference between the raw scores of our CADP patients and the HC in well-established neuropsychological tests such as SDMT, PASAT, TMT and RWT can be considered clinically relevant (SDMT: 41.38 vs. 54.65; PASAT: 18.5 vs 9.41; TMT-A: 42.31 vs. 26.72; RWTp: 20.56 vs. 26.25). One possible explanation is that in clinical practice, with regard to CADP, one usually concentrates on sensorimotor symptoms and the patients are very rarely asked by their treating physicians about any cognitive or emotional problems. Correspondingly, patients might focus predominantly on their progressive weakness or on sensory deficits while ignoring other symptoms. Furthermore, cognitive deficits may remain unnoticed unless a full neuropsychological testing is performed. Studies of peripheral diseases (CIDP/RA) have shown so far no success in detecting cognitive deficits when using only basic screening instruments such as MMSE [3,36].

The majority of our patients received intravenous immunoglobulin therapy (IVIGs), which sometimes may aggravate fatigue. However, two arguments suggest that the IVIG-related influence on fatigue (if any) is constrained to minimum or its confounding influence is accounted for. First, almost all (14 out of 16) subjects received the same treatment (IVIG). Furthermore, we included visual analogue scale fatigue measurements before and after the cognitive testing in our analyses as a covariate and there were no significant group differences—neither between the CADP and the healthy controls nor between the BBBd and BBBi groups—with regard to task-related fatigue change, which makes fatigue-driven group differences in cognitive performance less probable.

Neuropathic pain, which is a common problem among CADP patients, can also exhibit an influence on neuropsychological performance. However, only 3 out of 16 CADP patients reported neuropathic pain: one of them localized it in the calves, another one in the right gluteal area and only one in his hands. Based on this rather low prevalence of neuropathic pain in our sample and its localization predominantly in the lower extremities, we believe that its influence on the cognitive measures in the current study was not particularly high. Future studies should include more detailed examination of neuropathic pain (e.g. by employing pain-related questionnaires).

There are several limitations to our study. One of it is its limited sample size and heterogeneity of the included neuropathies. Larger studies with homogenous populations might be able to differentiate better between CADPs and demonstrate subtler effects. Furthermore, antibodies in serum as well as further CSF/serum markers for BBBd (ideally, not only CSF/serum albumin quotient but also additional markers such as for example S100 isoforms) should be measured prospectively to test for possible mechanisms of emergence of cognitive deficits in CADP patients. We analysed only retrospectively the CSF/serum quotients from the time of initial diagnosis which was in some cases several years before the cognitive testing. While the two groups (BBBd and BBBi) did not differ with regard to the time between the lumbar puncture and cognitive testing, future studies should ideally use a fully prospective design with a longitudinal approach and try to quantify baseline cognitive performance and BBBd at the same time as well as conduct follow-up cognitive measurements. Thus, due to the limitations of the current study, our suggestion that BBBd might be involved in the development of neuropsychological impairment in CADPs constitutes a rather tentative proposal for a possible mechanism, emerging from the association between retrospectively analysed clinical data and prospective cognitive testing.

Another limitation is the chosen definition of cognitive impairment. We decided to define cognitive impairment as test performance more than 1 SD below the respective reference mean as has been previously done in cognitive research with neurological patients [21,24,25,27–29]. While increasing the threshold to 1.5, 2 or more SD would have strengthened the validity of our results, this would have been a far more conservative cut-off and probably unsuitable for an exploratory study which aims at looking into a research question barely addressed before. The advantages of choosing lower SD scores as a threshold (higher power for the tested hypothesis) as well as disadvantages (grouping together different stages of cognitive impairment ranging probably from mild to severe and resulting in lower prevalence) are not confined to the population of CADP but have been extensively addressed also in other fields of cognitive research in neurology, e.g. in multiple sclerosis [37]. While our results are not able to differentiate clearly between mild, moderate and severe cognitive impairment distribution due to the small sample size, they are corroborated by the number of affected cognitive domains (44% of the patients exhibited cognitive impairment in at least two from five different cognitive domains) and the additional direct comparisons with healthy controls. A more precise look at the individual z-scores (Table 5) shows that most of the patients exhibited a rather mild to moderate manifestation of cognitive impairment (14 from 16 patients with at least one test score more than 1 SD below the reference mean; 6 from 16 patients with at least one test score more than 1.5 SD below the reference mean; 3 from 16 patients with at least one test score more than 2 SD below the reference mean). Thus, it would be an interesting question for a future study to test for more severe cognitive deficits in a larger population by defining a more conservative threshold for test failure.

While the gender distribution of our sample (3 females and 13 males) is not equal, it is very similar to the reported in the literature, where male/female ratio incidence and prevalence reach up to 4.4, indicating that males are overrepresented in immune-mediated neuropathies such as CIDP and MMN [38,39]. Because of the small sample size of the patients’ group and thus a very small number of females, we decided to not include the factor “gender” in the analysis for this exploratory study. While our main results are not based on the direct comparison between the patients’ and HC groups but rather on the neuropsychological performance of the patients with regard to the established test reference means, we believe that future studies should recruit larger patient samples and study the effect of gender on cognition in CADP patients.

Disease severity might be a confounding variable but at least in our sample this did not seem to be the case since the two subgroups (BBBd and BBBi), which differed with regard to the number of neuropsychological tests with below average performance did not differ with regard to their INCAT ODSS scores. Previous treatment effects on cognitive outcome might confound the results of cognitive testing, too. However, our sample size was too small to systematically test for difference between different medications.

Although MRI images/reports ruling out signs of central demyelination were available in approximately only half of the patients and there were no subjective reports about cognitive deficits or CNS comorbidities in the medical histories of our patients, conducting cranial magnetic resonance imaging (cMRI) in all subjects parallel to the cognitive testing would have strengthened this study methodologically by ensuring that there are no other concomitant pathologies.

Last but not least, it would have been a very interesting approach to test for an association between the cognitive deficits, BBB and peripheral antibodies usually detected in CADP (e.g. GM1-antibodies). Unfortunately, only a part of our sample has been tested for these antibodies during the diagnostic workup so we could not perform a reasonable analysis with regard to this question.

Notwithstanding the above-mentioned limitations, we believe that our study demonstrates an important and so far rather unknown clinical aspect of CADP, namely a mild to moderate impairment in a broad range of neuropsychological tests. These deficits seem to be associated with CSF evidence of BBBd, while the exact mechanism of this association remains unclear. Thus, neuropsychological symptoms and a possible CNS involvement might be considered in future studies of demyelinating neuropathies.
